# Electrode Coverage Optimization for Piezoelectric Energy Harvesting from Tip Excitation

**DOI:** 10.3390/s18030804

**Published:** 2018-03-07

**Authors:** Hailing Fu, Guangzhu Chen, Nan Bai

**Affiliations:** 1 Department of Aeronautics, Imperial College London, Exhibition Road, London SW7 2AZ, UK; 2 College of Nuclear Technology and Automation Engineering, Chengdu University of Technology, Chenghua District, Chengdu 610059, China; bn18328509248@163.com

**Keywords:** piezoelectric, electrode coverage, energy harvesting, cantilever beam, tip excitation, distributed parameter modelling

## Abstract

Piezoelectric energy harvesting using cantilever-type structures has been extensively investigated due to its potential application in providing power supplies for wireless sensor networks, but the low output power has been a bottleneck for its further commercialization. To improve the power conversion capability, a piezoelectric beam with different electrode coverage ratios is studied theoretically and experimentally in this paper. A distributed-parameter theoretical model is established for a bimorph piezoelectric beam with the consideration of the electrode coverage area. The impact of the electrode coverage on the capacitance, the output power and the optimal load resistance are analyzed, showing that the piezoelectric beam has the best performance with an electrode coverage of 66.1%. An experimental study was then carried out to validate the theoretical results using a piezoelectric beam fabricated with segmented electrodes. The experimental results fit well with the theoretical model. A 12% improvement on the Root-Mean-Square (RMS) output power was achieved with the optimized electrode converge ratio (66.1%). This work provides a simple approach to utilizing piezoelectric beams in a more efficient way.

## 1. Introduction

Energy harvesting, as an alternative energy source for low-power electronics, has prompted great research interest in the last decade. Three conversion mechanisms, i.e. piezoelectric, electromagnetic and electrostatic, have been extensively investigated for harvesting ambient kinetic energy from the environment [1,2]. Among these mechanisms, piezoelectric transduction has drawn significant attention due to its simplicity in structure, high energy density and good compatibility with micro-fabrication [3,4]. For piezoelectric energy harvesting, the most widely used structure is a cantilever-type structure with one or two piezoelectric layers mounted on a substrate layer. The strain generated on the beam by vibration or impact is converted to the electrical power using the piezoelectric effect. However, the output power from piezoelectric harvesters is still insufficient for powering most of the electronics, impeding the wide application of energy harvesting in self-powered sensing systems.

The low output power is mainly caused by the low power generation capability of piezoelectric transducers [5], and this capability is determined by several aspects, including material properties, cantilever shapes, structural parameters, etc. Bowen et al. provided a detailed review of piezoelectric materials [6]. The principles and material characteristics were discussed in detail for different types of piezoelectric materials. Goldschmidtboeing and Woias studied the impact of the cantilever shape on the energy harvesting capability [7]. This study conclued that triangular-shaped beams were more effective than rectangular-shaped beams in terms of the maximum tolerable excitation amplitude and the maximum output power. Park and Kwak [8] optimized the beam shape for external excitation at the beam’s free end. This work found that a cantilever-type beam with a wide free tip has better performance than the simple rectangular cantilever or the well-known tapered cantilever. Dhakar et al. demonstrated a composite cantilever with a proof mass at the free end [9]. The beam was comprised of a piezoelectric bimorph and a polymer beam. The polymer beam was mechanically connected to the piezoelectric beam at its free end. This composite beam exhibited a higher conversion capability and a wider bandwidth than a standalone bimorph.

The size and shape of the piezoelectric transducer also affect the performance of harvesters. Friswell and Adhikari investigated the influence of the length and width of the piezoelectric layer of a rectangular beam on the power output [10]. The width can be varied along the beam length in the optimization. The results show that the transducer shape alters both the capacitance and the coupling factor. Maximum power output can be obtained for a certain length of the transducer layer. Patel et al. developed a comprehensive theoretical model for analysing piezoelectric cantilever beams with varying length and thickness of the piezoelectric layer [11]. These two parameters proved to be significant for increasing the output power. Zhou et al. investigated flexible longitudinal zigzag energy harvesters for the purpose of enhancing energy harvesting from low-frequency and low amplitude excitation [12]. Both theoretical and experimental results illustrated the capability of harvesting low-frequency and low-amplitude energy from multi-directions. In addition to the cantilever structure, Liu et al. presented a nonlinear wideband harvester architecture by clamping the cantilever beam with a curve fixture [13]. The applied fixture allowed the harvester to demonstrate a wide operating bandwidth.

In addition, electrode coverage is another important factor for piezoelectric harvester design. For piezoelectric transducers, along with the piezoelectric layer, there are normally two additional metal layers as electrodes for transferring the electrons from the piezoelectric material to the subsequent load circuits [14]. Using partial or segmented electrodes provides a method to improve the performance of piezoelectric energy harvesters. For the simple rectangular cantilever beams, they can operate at different vibration modes with bipolar strain distributions on the same plane (parallel to the beam’s neutral layer). Segmented electrodes are necessary in this scenario to collect the electrical energy generated from bipolar strains. Erturk et al. studied the effect of strain nodes and electrode configuration for harvesters operating in high vibration modes [15]. A cancellation effect was observed and tested using continuous electrodes, and increased output power was obtained using segmented electrodes divided based on the position of strain nodes. Marqui et al. proposed an airflow energy harvester using the cantilever beam [16]. Segmented electrodes were fabricated in the beam width direction. The energy from torsional motion was harnessed, and the segmented electrodes were adopted to address the strain complication caused by the torsional motion. Pineirua et al. studied the influence of the arrangement of the piezoelectric electrodes along the plate’s surface on the power generation capability of a fluttering piezoelectric harvester [17]. In the case of a single electrode and small values of the mass ratio, a greater amount of energy can be harvested when positioning the electrodes on the downstream half of the device. At a higher mass ratio, more than one optimal positioning are found and using a larger number of electrodes improves the output. Krishnasamy and Lenka built a theoretical mode to study the charge cancellation effect in cantilever beams operating at high vibration modes [18]. Solutions of using segmented electrodes were proposed to prevent the cancelling effect.

For micro-harvesting devices, the resonant frequency for high order vibration modes is normally much higher than the frequency of ambient vibrations in the environment, which are characterized as low frequency and broadband [19,20]. Therefore, energy harvesters using cantilever structures typically operate at their first vibration mode. In this mode, the strain distribution varies linearly along the length direction from the maximum at the root to zero at the free end, but the electric potential is even when electrodes are fully covered on the piezoelectric layers. Energy is dissipated by the movement of electrons from the high potential region to the low potential region. In order to minimize this energy dissipation, Mark et al. provided a simplified analysis for the electrode coverage of cantilever beams under base excitation [21]. The piezoelectric beam is considered as a capacitor in this analysis, which means the mechanical behaviour was ignored. Cho et al. developed a theoretical model for a simply-supported membrane-type piezoelectric energy harvester [22]. The maximum electromechanical coupling coefficient was obtained with an electrode coverage of 42%, but the strain distribution in this case is different from that of cantilever beams. Du et al. provided an analysis of the electrode coverage for cantilever beams, showing the optimal coverage percentage of 44% [23], but the external force in this study is a uniformly distributed force along the beam length direction, which is different from the normally used base excitation [24] or tip excitation [25,26].

In this paper, we present a theoretical model to analyze the influence of electrode coverage on the electromechanical behaviour of the piezoelectric harvester under tip excitation. Compared with base excitation, the tip excitation method (also known as “Frequency Up-Conversion”) demonstrates broadband capability by beam plucking using magnetic force [27,28] or direct impacts [29,30]. This method has been extensively adopted and studied in recent years for different energy harvesting scenarios, including harvesting from human motion [31], airflow [32], and rotational sources [33], and this method has been regarded as a better way for piezoelectric energy harvesting in many practices. In this study, the harvester is plucked by a magnetic force at the tip and operates at its fundamental vibration mode. A theoretical model considering the variation of the electrode converge ratio is established to study the system dynamics. The capacitance, output power and optimal load resistance are studied for varying lengths of electrodes. The theoretical results are then verified experimentally to show the capability of the model to optimize the electrode coverage. This work provides a simple way to design and use piezoelectric transducers more effectively.

## 2. Theoretical Modelling of Piezoelectric Harvester with Varying Electrode Coverage

### 2.1. Harvester Design and Configuration

**Figure 1:**
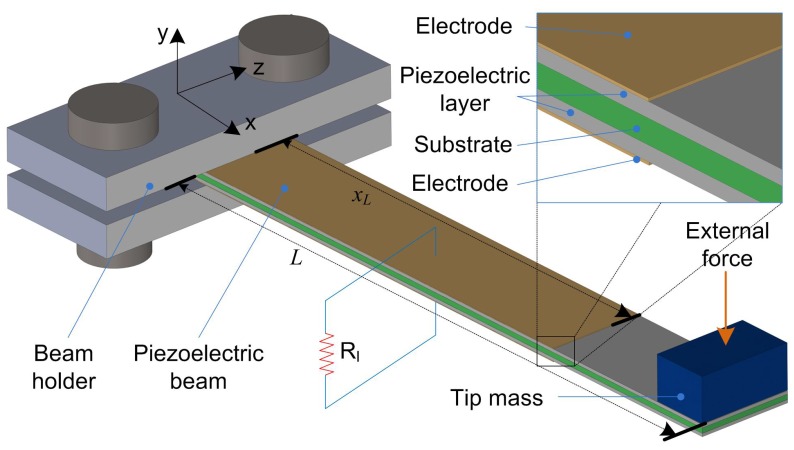
Schematic diagram of a general bimorph piezoelectric energy harvester with a substrate and two electrodes.

Figure 1 shows the typical configuration of a piezoelectric energy harvester using a cantilever-type beam under tip excitation. In this figure, there are two piezoelectric layers, and they can be connected in series or parallel according to the polarization direction of the piezoelectric material. In this study, these layers are connected in series. An external force is applied on the beam’s free end as the tip excitation force. For tip excitation, the force normally plucks the beam in a short time frame in one excitation cycle and then releases the beam, allowing the beam to vibrate freely at resonance before the subsequent excitation force appears (frequency up-conversion). Mechanical impacts or magnetic force are typically adopted for tip excitation. The piezoelectric material is deposited on the whole beam area, but the electrode length $x_{L}$ is designed to be changeable in order to optimize the performance of the harvester. A resistive electrical load is connected to the electrodes for power extraction.

**Figure 2:**
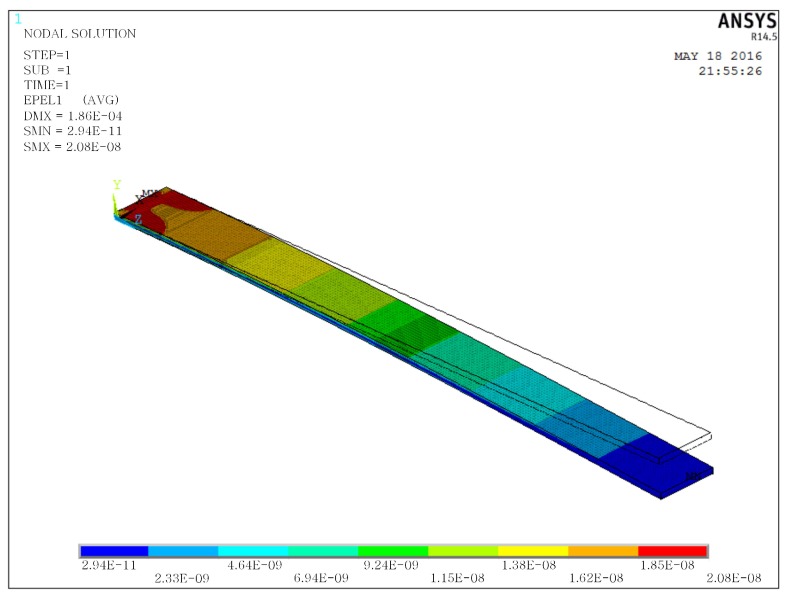
Example of strain distribution of a cantilever beam under tip excitation.

Figure 2 is a typical example of the strain distribution of a cantilever beam under tip excitation. The strain decreases linearly from the fixed end to the free end in the beam length direction. Therefore, the power generating capability weakens from the fixed to the free as well, as the output voltage from the piezoelectric beam can be assumed to be proportional to the stress on the piezoelectric material [34]. This means the voltages generated by the tip excitation on the piezoelectric material also decrease from the fixed end to the free end. However, when electrodes cover on the whole beam, the electric potential on the whole area is uniform, which means charges have to move from the high potential area (fixed end) to the low potential area (free end). Energy is dissipated by this charge movement. Using partial electrodes can reduce the energy lose from charge movement, but the total charges that can be obtained are reduced as well. Hence, there is a trade-off between the total charge collected and the energy dissipated by charge movement. It is worth studying the optimal electrode coverage ratio to achieve the best energy harvesting performance.

### 2.2. Modelling of Harvester with Variable Electrode

The variation of the electrode coverage not only affects the electrical behaviour of the harvester, but also its mechanical performance, namely the vibration response. Therefore, a complete model considering both the mechanical and electrical fields are needed to optimize the design.

The current output of the harvester can be obtained from the integral form of Gauss’s Law [35], which is

(1) $$\begin{array}{c} i\left(t\right)=\frac{dQ(t)}{dt}=\frac{d}{dt}\int_{A}\left(D\cdot n\right)dA=\frac{v(t)}{R_{l}}, \end{array}$$

where *Q* is the electric charge on the electrodes, D is the vector of electric displacement in the piezoelectric layer, n is the unit outward normal, and the integration is performed over the electrode area *A*. v(t) is the output voltage across the resistive load $R_{l}$. The electric displacement can be calculated using the piezoelectric constitutive equations, and the vector D·n can be reduced to the following scalar expression, as the beam operates in the $d_{31}$ mode:

(2) $$\begin{array}{c} D\cdot n=D_{3}={\bar{e}}_{31}S_{1}^{p}+{\bar{\varepsilon }}_{33}^{S}E_{3}, \end{array}$$

where ${\bar{e}}_{31}$ is the piezoelectric constant, $S_{1}^{p}$ is the strain component on the piezoelectric layer, ${\bar{\varepsilon }}_{33}^{S}$ is the dielectric constant and $E_{3}$ is the electric field component, which can be written as $E_{3}=-v(t)/\left(2h_{p}\right)$. $h_{p}$ is the thickness of the piezoelectric layer.

The axial strain mid-way through each piezoelectric layer is proportional to the curvature of the beam at that point:

(3) $$\begin{array}{c} S_{1}^{p}=-h_{pc}\frac{\partial^{2}\omega \left(x,t\right)}{\partial x^{2}}, \end{array}$$

where ω(x,t) is the transverse displacement of the beam, $h_{pc}$ is the distance between the neutral axis and the center of the piezoelectric layer.

The electrical equation of piezoelectric beams are then obtained from Equation (1) as

(4) $$\begin{array}{c} {\bar{e}}_{31}bh_{pc}\int_{0}^{x_{L}}\frac{\partial^{3}\omega \left(x,t\right)}{\partial x^{2}\partial t}dx+\frac{{\bar{\varepsilon }}_{33}^{S}bx_{L}}{2h_{p}}\frac{dv(t)}{dt}+\frac{v(t)}{R_{l}}=0, \end{array}$$

where $x_{L}$ is the length of the electrodes.

**Figure 3:**
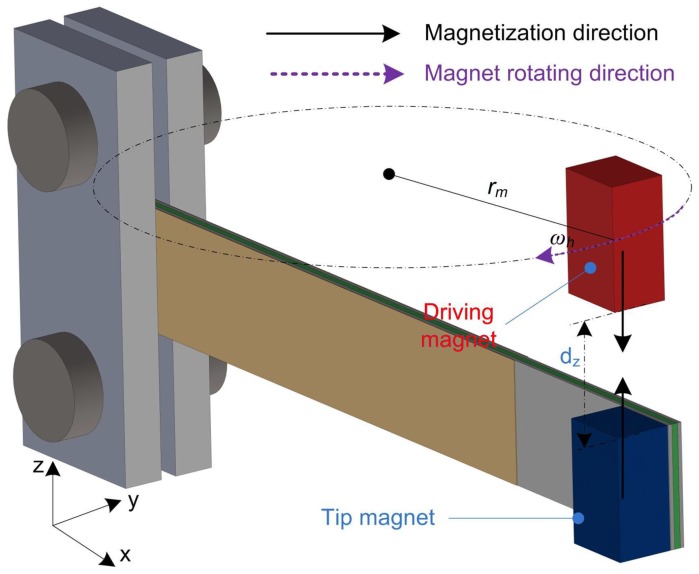
Strain distribution of a cantilever beam under tip excitation.

The strain distribution can be deduced from the vibration response of the cantilever under tip excitation. In terms of the excitation mechanism, a magnetic-force plucking method is adopted in this analysis, as this is the typical solution for the tip excitation method, and the force model has been verified by several researchers [20,31]. The schematic showing the operating mechanism is depicted in Figure 3. There is a driving magnet rotating over the piezoelectric beam. The rotational motion can be provided by ambient rotation sources, such as moving vehicles, miniature turbines or pendulums. The beam is plucked by the magnetic force once per rotation cycle. Due to the high natural frequency of the beam and the low excitation frequency of the driving magnet, the beam vibrates freely after each excitation at its natural frequency for a few cycles before the second excitation force appears. The rotational radius of the driving magnet is $r_{m}$. The rotational speed is $\omega_{h}$. The gap between the magnets in the *z*-axis is $d_{z}$.

Erturk and Inman built a distributed-parameter model for piezoelectric beams under base excitation [36]. In our study, adaptation has been made to replace the base excitation to the tip excitation case. A direct tip force is introduced to replace the base acceleration. The mechanical equation of the beam motion under tip excitation can be written as

(5) $$\begin{array}{c} \mathrm{YI}\frac{\partial^{4}\omega \left(x,t\right)}{\partial x^{4}}+c_{s}I\frac{\partial^{5}\omega \left(x,t\right)}{\partial^{4}x\partial t}+c_{d}\frac{\partial \omega (x,t)}{\partial t}+m\frac{\partial^{2}\omega \left(x,t\right)}{\partial t^{2}}\\ -\vartheta v\left(t\right)\left[\frac{d\delta (x)}{dx}-\frac{d\delta (x-L)}{dx}\right]=F_{tip}^{y}\left(t\right)\delta \left(x-L\right), \end{array}$$

where *YI* is the bending stiffness, $c_{s}I$ is the internal damping, $c_{d}$ is the viscous deformation damping, *m* is the mass per unit length of the beam, δ(x) is the Dirac delta function, ϑ is the piezoelectric coupling term in physical coordinates, $F_{tip}^{y}\left(t\right)$ is the tip force on the piezoelectric beam and *L* is the length of the beam. Details on the solution to this equation are referred to Ref. [36].

Due to the three-dimensional distribution of the driving magnet and the tip magnet, a 3D theoretical model is needed to calculate the magnetic force. Akoun and Yonnet established a reliable theoretical model for calculating 3D magnetic force between cuboidal magnets [37]. The magnetic force on the tip magnet in the beam bending direction is

(6) $$\begin{array}{c} F_{tip}^{y}=\frac{J\cdot J^{\prime }}{4\pi \mu_{0}}\sum_{i=0}^{1}\sum_{j=0}^{1}\sum_{k=0}^{1}\sum_{l=0}^{1}\sum_{p=0}^{1}\sum_{q=0}^{1}{\left(-1\right)}^{i+j+k+l+p+q}\cdot \phi_{y}, \end{array}$$

where *J* and $J^{\prime }$ are the magnetizations of the magnets, $\mu_{0}$ is the magnetic constant, and $\phi_{y}$ is a function of the magnet dimensions and their gaps in three axes. The function is given by

(7) $$\begin{array}{c} \phi_{y}=\frac{1}{2}\left(U_{ij}^{2}-W_{pq}^{2}\right)\ln\left(r-V_{kl}\right)+\frac{1}{2}rV_{kl}+U_{ij}W_{pq}\tan^{-1}\left(\frac{U_{ij}V_{kl}}{rW_{pq}}\right)+U_{ij}Vkl\ln\left(r-U_{ij}\right), \end{array}$$

where

(8) $$\begin{array}{c} \left\{\begin{array}{c} U_{ij}=d_{x}+{\left(-1\right)}^{j}A-{\left(-1\right)}^{i}a,\\ V_{kl}=d_{y}+{\left(-1\right)}^{l}B-{\left(-1\right)}^{k}b,\\ W_{pq}=d_{z}+{\left(-1\right)}^{q}C-{\left(-1\right)}^{p}c,\\ r=\sqrt{U_{ij}^{2}+V_{kl}^{2}+W_{pq}^{2}}. \end{array}\right. \end{array}$$

These lengths, $U_{ij}$, $V_{kl}$ and $W_{pq}$ correspond to the distance between the cube corners and their projections on the axes. The parameters *i*, *j*, *k*, *l*, *p* and *q*, are equal to 0 or 1 according to the specific corner. $d_{x}$, $d_{y}$ and $d_{z}$ are the gaps between two magnets in three axes. Assuming the rotational frequency of the host is $\omega_{h}$, the gaps can then be expressed as

(9) $$\begin{array}{c} \left\{\begin{array}{c} d_{x}=r_{m}+d_{x0}-r_{m}\cos\left(\omega_{h}t-\alpha_{m0}\right),\\ d_{y}=r_{m}\sin\left(\omega_{h}t-\alpha_{m0}\right)-\omega \left(L,t\right),\\ d_{z}=d_{z0}, \end{array}\right. \end{array}$$

where $d_{x0}$ and $d_{z0}$ are the initial gaps along the *x*-axis and *z*-axis when the rotor is static, with the driving magnet’s angular position $\alpha_{m0}=0$, ω(L,t) is the tip displacement of the piezoelectric beam in the y direction, namely the beam bending direction, and $r_{m}$ is the rotational radius of the driving magnet. For further details, please refer to [37].

Combining Equations (4) and (5), we can get the electromechanical performance of the beam for varying electrode coverage.

### 2.3. Analysis of Electromechanical Dynamics

The theory introduced above is solved using Matlab/Simulink (R2017a, MATrix LABoratory, Natick, MA, UNITED STATES). The parameters of the harvester are listed in Table 1. The piezoelectric material is M1100 from Johnson Matthey Piezo Products (Redwitz, Germany). In this study, the length $x_{L}$ of the electrodes is variable, and the influence of the variable electrode coverage on the electromechanical performance is analyzed.

**Figure 4:**
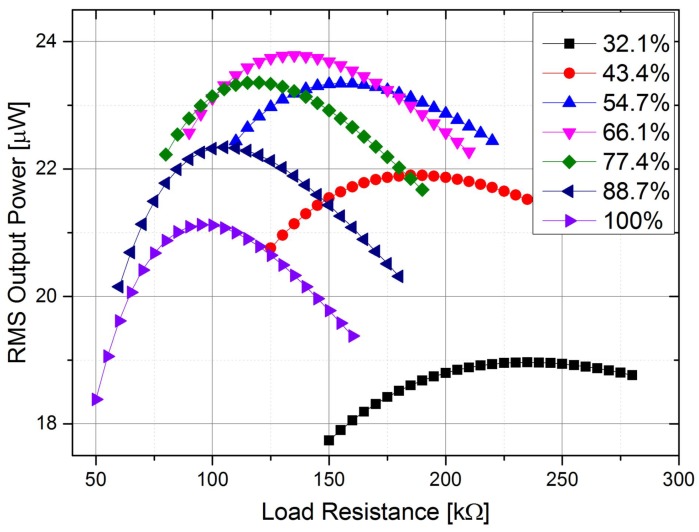
RMS output power versus load resistance for harvesters with different electrode coverage ratios.

Figure 4 shows the Root-Mean-Square (RMS) output power against load resistance for different electrode coverage ratios. The coverage ratio increases with a 11.3% sub-step. The optimal load resistance decreases from 235 kΩ to 95 kΩ when the electrode coverage increases from 32.1% to 100%. The maximum output power for different coverage ratios changes as well. With the electrode coverage of 66.1%, the maximum output power is the highest (23.8 μW). Compared with the output power with full electrode coverage (21.1 μW), the output power with the coverage of 66.1% has a 12.8% improvement. However, when the coverage ratio keeps decreasing, the output power reduces because of the decreased charge generation. In addition, with the increase of the coverage ratio, the output becomes more sensitive to the variation of the load resistance. This can be identified by comparing the curves for different ratios. The curve for the 100% coverage is much sharper than the one with the 32.1% coverage.

**Figure 5:**
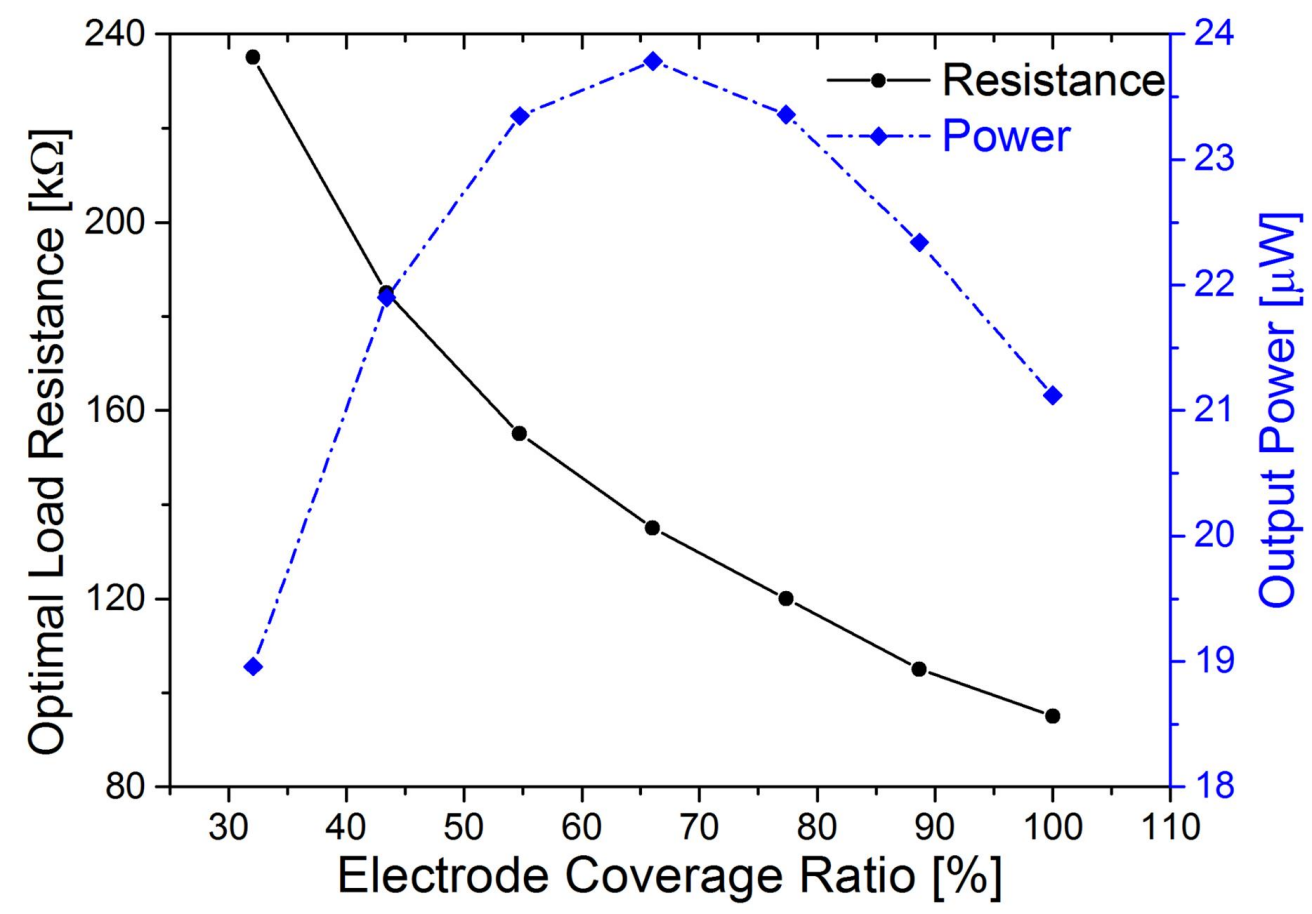
The variation of the optimal load resistance and the corresponding RMS output power against electrode coverage ratio.

Piezoelectric energy harvesters can be regarded as a current source in parallel with the electrode capacitance $C_{p}$ [38]. The internal capacitance varies with the electrode length ($C_{p}=\varepsilon \cdot b\cdot x_{L}/\left(2h_{p}\right)$). Therefore, the optimal load resistance increases with decrease of the electrode length ($R_{opt}=1/\left(\omega_{n}C_{p}\right)$). Figure 5 depicts the variation of the optimal load resistance and the output power against electrode coverage ratio. The resistance shows an inverse relationship with the electrode coverage ratio, which is proportional to the capacitance. With the increase of the electrode coverage ratio, the output power increases first and decreases subsequently after the ratio is over 66%, showing the trade-off between the total collected charges and the energy dissipated by charge movement.

**Figure 6:**
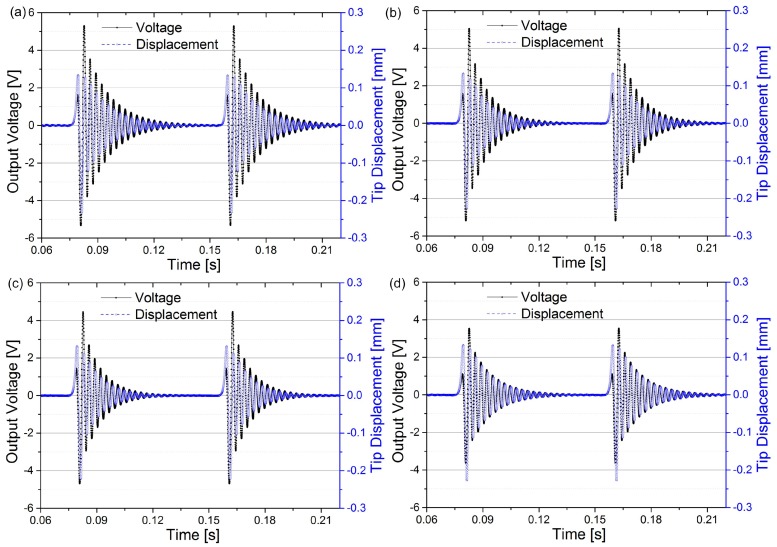
The variation of beam tip displacement and output voltage for different electrode coverage ratios with their optimal resistive loads connected. (**a**) 32.1% (235 kΩ); (**b**) 43.4% (185 kΩ); (**c**) 66.1% (135 kΩ) and (**d**) 100% (95 kΩ). The driving magnet rotates at 12.5 Hz.

Figure 6 shows the variation of the beam tip displacement and the output voltage for different electrode coverage ratios. The driving magnet plucks the piezoelectric beam at 12.5 Hz, and the optimal resistive loads are connected with the harvester with different coverage ratios, respectively. This figure shows that the electrode coverage has a significant influence on the electrical behaviour of the harvester. The optimal load resistance decreases from 235 kΩ to 95 kΩ when the electrode coverage increases from 32.1% to 100%, and the peak output voltage decreases from 5.3 V to 3.5 V. The improved output voltage for partial electrode coverage situations is also beneficial to the current rectification process, which means that the energy conversion efficiency can be also improved by the increased output voltage.

The tip displacement, however, stays unchanged for different coverage ratios, which indicates that the electrode coverage has a limited influence on the mechanical behaviour of the harvester. Furthermore, it also indicates that the electrical energy collected from the vibrating piezoelectric beam is marginal compared with the mechanical energy in the beam. Therefore, the improved electrical output power is not indicated on the tip displacement curves. In order to enable the piezoelectric energy harvester to work more effectively, other methods should be adopted to enhance the electrical damping of the system, such as the pre-biasing method [39], the flipping-capacitor rectifier topology [40] or the synchronized switch harvesting interface circuits [41] to increase the electrical damping.

## 3. Experimental Set-Up

**Figure 7:**
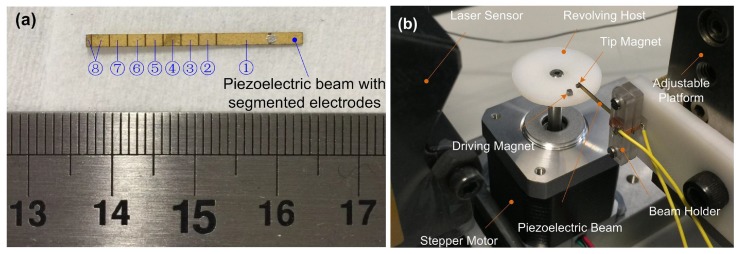
Piezoelectric beam with segmented electrodes (**a**) and experimental set-up (**b**).

In order to verify the theoretical analysis, an experimental validation was conducted for piezoelectric energy harvesters with different electrode coverage ratios. The piezoelectric beam is a commercial product from Johnson Matthey. The varying electrode coverage was achieved by dividing the electrodes using laser cutting. The segmented beam is shown in Figure 7a. The beam was divided into seven segments. During tests, the right end of the beam was fixed on a beam holder shown in Figure 7b. A magnet was mounted at the beam’s left end as the tip magnet. The generated charges were transferred into the subsequent circuits by wires on the beam holder. In the beginning of the test, only segment 1 (representing 32.1% electrode coverage) was connected with the resistive load. Afterwards, each segment was connected with segment 1 by conductive adhesive sequentially. By adding one segment, the coverage ratio increased 11.3%.

The experimental set-up is demonstrated in Figure 7b. The beam is installed on an adjustable platform that is capable of precisely controlling the position of the piezoelectric beam in three directions. A high speed stepper motor (Phidgets 3303, Calgary, AB, Canada) with the step angle of 1.8${}^{\circ }$ is placed underneath the beam with a revolving plate mounting on the motor’s shaft. The motor is driven by a bipolar motor control circuit (Phidgets 1067), which has a position resolution of 116 step. The acceleration is also programmable to achieve any desired rotational speed. A magnet is mounted on the revolving plate as the driving magnet. Magnetic plucking is formed by the driving magnet and the tip magnet at the beam’s free end. The relative position of the magnets is regulated by the adjustable platform. A laser displacement sensor is also adopted in the experiment to measure the beam tip displacement.

## 4. Results and Discussion

**Figure 8:**
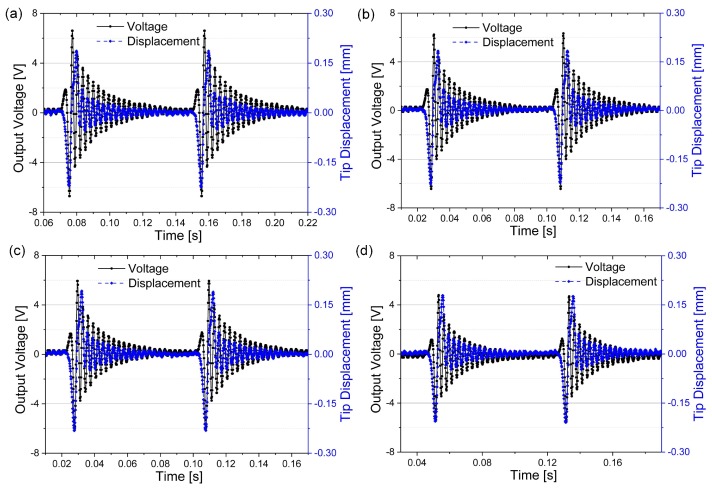
Variation of beam tip displacement and output voltage for different electrode coverage ratios with their optimal resistive loads connected. (**a**) 32.1% (200 kΩ); (**b**) 54.7% (150 kΩ); (**c**) 66.1% (130 kΩ) and (**d**) 100% (100 kΩ). The driving frequency was 12.5 Hz.

Figure 8 depicts the time domain response of the output voltage and beam tip displacement for four different electrode coverage ratios. The output voltage was measured from the connected load resistors. The optimal load resistance was tested and connected for different converge ratios. From the displacement curves, the amplitude almost remained unchanged, which indicates the low electromechanical coupling of the piezoelectric beam. This trend is the same as the theoretical results as shown in Figure 6. For the output voltage, the value deceases with the increase of electrode coverage from 6.6 V for 33.1% coverage to 4.8 V for 100% coverage, but with larger electrode converge, the optimal load resistance decreases as well (200 kΩ for 33.1% and 100 kΩ for 100%). Compared to the theoretical results in Figure 6, the experimental results have a close match, and the difference between the amplitude of output voltage could be caused by the difference of the excitation magnetic forces used between the theoretical and experimental results. In general, the experimental results verifies the theoretical results in Figure 6.

**Figure 9:**
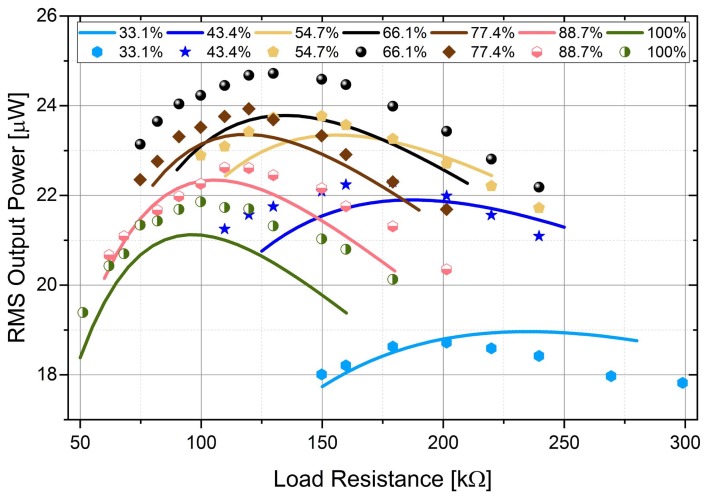
RMS output versus load resistance for different electrode coverage ratios. The solid lines and marks are theoretical and experimental results, respectively. Identical colors are applied to the same electrode coverage ratio.

In order to get the optimal electrode coverage ratio for the maximum output power, piezoelectric beams with different electrode coverage ratios were tested for different load resistance. Figure 9 shows the RMS output power of the segmented harvester against load resistance. The RMS output was calculated using the voltage output (as shown in Figure 8) for a particular load resistance within one excitation cycle. The variation of the maximum RMS output power and the optimal load resistance for different coverage ratios is verified experimentally. The optimal load resistance decreases with the increase of the ratio. The maximum RMS output power (24.6 μW) is achieved when the coverage ratio is 66.1% with the optimal load resistance of 130 kΩ. There is a 11.9% improvement against the output power (21.9 μW) of the harvester with full electrode coverage.

The theoretical and experimental results are compared in Figure 9 as well. These results do not perform a perfect match, but the general trends are the same for different coverage ratios. Possible reasons for the mismatch could be the differences in the magnetic excitation force amplitudes, inaccurate structural and material parameters used in the theoretical calculation, or experimental errors, including imperfect clamping conditions of the piezoelectric beam, errors from the dimensions of the segmented electrodes, or electrode damage caused by laser cutting, etc. Overall, the experimental results illustrate the variation of the performance of the piezoelectric beam for different electrode coverage ratios and also verify the theoretical analysis.

**Figure 10:**
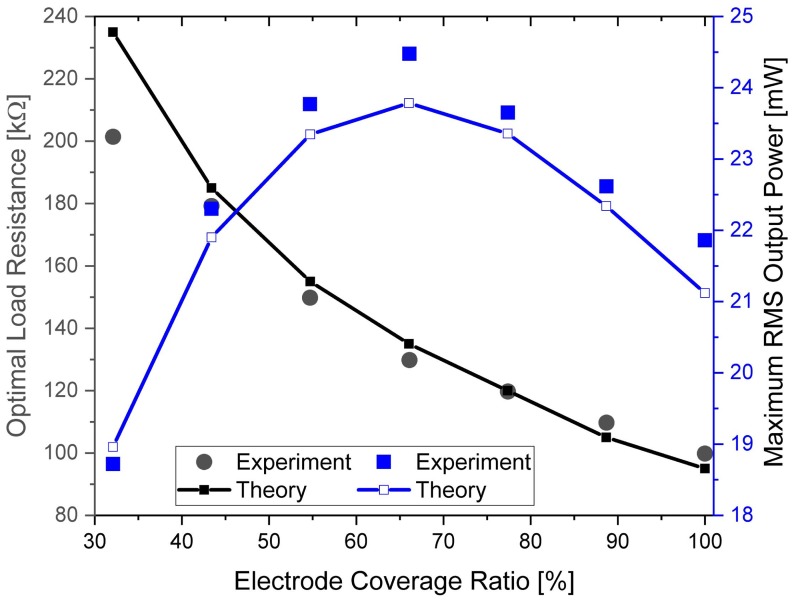
Optimal load resistance and corresponding maximum output power against electrode coverage ratio. The blue line is the output power, and the black line is the optimal load resistance.

Figure 10 depicts the optimal load resistance and corresponding maximum RMS output power of the harvester against coverage ratios. The optimal load resistance also shows an inverse relationship with the coverage ratio, compared to that in Figure 5. The resistance decreases from 200 kΩ to 100 kΩ with the increase of electrode coverage from 32.1% to 100%. Again, the experimental results are close to the theoretical analysis, and the advantage of the partial electrode converge method is illustrated for improving the power generating capability of piezoelectric harvesters.

## 5. Conclusions

In this paper, the electrode coverage ratio is investigated in order to improve the output power of piezoelectric energy harvesters under tip excitation. A distributed-parameter model was built for a bimorph piezoelectric cantilever beam with consideration of the electrode coverage. The influence of the electrode coverage on beam vibration, internal capacitance, optimal load resistance and output power is analysed theoretically and then verified experimentally.

The optimal coverage was found to be 66.1% with a 12.8% improvement of the output power in theoretical analysis. The optimal load resistance increases with the decrease of the electrode coverage ratio, as the internal capacitance decreases. The output voltage, however, increases with decrease of the electrode coverage ratio, which means that the performance of the harvester can be improved with the optimized electrode design. The tip displacement remains the same for the harvester with different coverage ratios working at the optimal load condition. It indicates that, compared with the mechanical energy in the vibrating beam, the extracted electrical energy is marginal. Other mechanisms to improve the electrical damping are needed to further improve the performance of piezoelectric harvesters.

In experiments, a piezoelectric beam was cut into seven segments using laser machining. The beam tip displacement and output voltage were measured for the harvester with different coverage ratios. The experimental results verify the theoretical analysis from both the mechanical and the electrical perspective, showing that partial electrode coverage is beneficial to enhancing the performance of energy harvesting using piezoelectric cantilever beams. This study provides a simple and effective way to improve the power generating capability of piezoelectric energy harvesters under tip excitation.

## Figures and Tables

**Table 1:** Design parameters of the piezoelectric turbine.

Symbol	Description	Value
L×b	Beam length and width	26.5mm×1.5mm
$h_{p}$	Thickness of piezo layer	0.1 mm
$h_{s}$	Thickness of substrate	0.1 mm
$r_{m}$	Magnet rotation radius	12 mm
*a × b × c*	Driving magnet size	0.5×0.5×0.5mm
*A × B × C*	Tip magnet size	0.5×0.5×0.5mm
$d_{z0}$	Initial gap in *z*-axis	3.2 mm
*J*	Magnetization of magnets	1.17 T
$\rho_{m}$	Density of magnets	$7400\;\mathrm{kg}/m^{3}$
${\bar{e}}_{31}$	Piezoelectric constant	−22.2V·m/N
$d_{31}$	Piezoelectric charge constant	−315 m/V
$\rho_{p}$	Density of piezoelectric material	7700kg/m${}^{3}$
$\rho_{s}$	Density of substrate material	1500kg/m${}^{3}$
*Y*	Young’s modulus of substrate	140GPa
$\epsilon_{r33}^{T}$	Relative permittivity constant	4500
